# Harnessing dual applications of a novel ascomycetes yeast, *Starmerella cerana* sp. nov., as a biocatalyst for stereoselective ketone reduction and biosurfactant production

**DOI:** 10.3389/fbioe.2023.1264826

**Published:** 2023-10-24

**Authors:** Sachin Kumar, Nitish Kumar Verma, Sandal Deep Basotra, Divya Sharma, G. S. Prasad, Mani Shankar Bhattacharyya

**Affiliations:** ^1^ Microbial Type Culture Collection (MTCC), CSIR-Institute of Microbial Technology (IMTECH), Chandigarh, India; ^2^ Biochemical Engineering Research and Process Development Centre (BERPDC), CSIR-Institute of Microbial Technology (IMTECH), Chandigarh, India

**Keywords:** phylogeny, *Starmerella cerana*, honey bee surface, novel taxa, bumblebee, biocatalysis, asymmetric reduction, sophorolipid

## Abstract

**Introduction:** New bioresources for catalytic application and fine chemical synthesis are the need of the hour. In an effort to find out new biocatalyst for oxidation-reduction reaction, leading to the synthesis of chiral intermediates, novel yeast were isolated from unique niche and employed for the synthesis of value added compounds.

**Methods:** To determine the genetic relatedness of the isolated strain, HSB-15^T^, sequence analysis of the internal transcribed spacer (ITS) and D1/D2 domains of the 26S rRNA gene sequence was carried out. The distinctive features of the strain HSB-15^T^ were also identified by phenotypic characterization. The isolated strain HSB-15^T^ was employed for the reduction of selected naphthyl ketones to their corresponding alcohols and a biosurfactant was isolated from its culture broth.

**Results:** The analysis of the ITS and D1/D2 domains of the 26S rRNA gene revealed that strain HSB-15^T^ is closely related to the type strain of *Starmerella vitae* (CBS 15147^T^) with 96.3% and 97.7% sequence similarity, respectively. However, concatenated sequences of the ITS gene and D1/D2 domain showed 94.6% sequence similarity. Phenotypic characterization indicated significant differences between strain HSB-15^T^ and its closely related species and consequently, it was identified as a novel species, leading to the proposal of the name *Starmerella cerana* sp. nov. The strain was able to reduce selected naphthyl ketones to their corresponding alcohols with remarkable efficiency, within a 12-hours. The strain HSB-15T also produced a surfactant in its culture broth, identified as sophorolipid upon analysis.

**Discussion:** The study explored the potential of the novel strain, HSB-15T, as a whole-cell biocatalyst for the reduction of naphthyl ketones to their corresponding alcohols and also reports its capability to produce sophorolipid, a biosurfactant, in its culture broth. This dual functionality of HSB-15T both as biocatalyst and biosurfactant producer enhances its applicability in biotechnology and environmental science.

## 1 Introduction

Process innovation and recent developments in the pharmaceutical industry have increased the production capacity of drugs and pharmaceuticals, making them accessible to the general public. However, these industries are often criticized for creating environmental pollution (Naidu et al., 2021). Thus, environmental concerns, the need for better process yield and ease, and the generation of a lower quantity of waste have pushed the scientific community toward the development of greener, environmentally benign processes for the pharmaceutical industry (Woodley, 2008). Biocatalytic synthesis of pharma-chemicals and intermediates is one of the ways toward achieving environmentally benign technologies. Yeasts are known for their capability to carry out biocatalytic conversions relevant to the pharmaceutical and fine chemical industries. However, finding novel yeast carrying out such conversions opens up a new window for creating new biocatalysts with better substrate scope.

Flowers are a major source of yeast biodiversity. Flower-associated yeast communities utilize a wide variety of sugar-enriched substrates (found in the nectar) that serve as a highly enriched medium for the survival of yeast communities (Brysch-Herzberg, 2004; Morais, Pagnocca, and Rosa, 2006; Belisle et al., 2014; Sipiczki, 2015a). The yeast genera commonly found in nectar and floral surfaces include *Metschnikowia*, *Cryptococcus*, *Rhodotorula*, *Clavispora*, *Kodamaea*, *Debaryomyces*, *Sporobolomyces*, *Hanseniaspora*, *Candida*, *Papiliotrema*, *Wickerhamiella*, and *Starmerella* (Klaps, Lievens, and Álvarez-Pérez, 2020). On the other hand, the yeast communities found in bees and their habitats are largely members of a clade centered around the genus *Starmerella* (Rosa et al., 2003; Teixeira et al., 2003). Species of the genus *Starmerella* isolated from bees and other sources are mentioned in Table 1. Members of the genera *Rhodotorula*, *Debaryomyces*, and *Candida* are known to have oxido-reductive biocatalytic potential; however, members of the genera *Metschnikowia*, *Wickerhamiella*, and *Starmerella* are known to have the capacity of producing biosurfactants. In fact, yeasts from the genus *Starmerella* have been explored as an industrially relevant microorganism, typically for the production of a versatile biosurfactant, sophorolipid (SL), which is known to have a wide range of applications in the healthcare and cosmetic industries (Kurtzman et al., 2010; Morya et al., 2013). Therefore, our objective was to find honey bee-associated yeasts that can carry out biocatalytic functions and produce biosurfactants.

**TABLE 1:** Yeast strains used for the construction of the phylogenetic tree in this study.

Organism name	ITS ACCESSION no.	D1/D2 ACCESSION no.	CBS no.	Source of isolation	Country	ITS size (bp)	D1/D2 size (bp)	Sequence depositor
*Starmerella* sp. *MOM_864*	HG421428			Floral nectar	Spain	895		Mittelbach et al., (2015)
*Starmerella* sp		MG564473		Flower	Brazil		441	Santos,A.R.
*Starmerella aceti*	KF271437		13,086	Fungus garden	Brazil	434		Melo et al., (2014)
*Starmerella aceti*		KF247224	13,086	Fungus garden	Brazil		480	Melo et al., (2014)
*Starmerella* sp.	KC776265		12811T	Melipona quinquefasciata	Brazil	927		Daniel et al., (2013)
*Starmerella apicola*	EU926482		2868T	Floricolous insects		459		Lachance et al., (2010)
*Starmerella apicola*		U45703	2868T	NA	NA		480	Kurtzman and Robnett (1998)
*S*tarmerella sp.	KU128719		14173T	NA	NA	404		Hui,F.L.
*Starmerella* sp.		KU128728	14173T	NA	NA		483	Hui,F.L.
*Starmerella* sp.	KU128715		14178T	NA	NA	411		Hui,F.L.
*Starmerella* sp.		KU128732	14178T	NA	NA		482	Hui,F.L.
*Starmerella* sp.	KU128716		14172T	NA	NA	415		Hui,F.L.
*Starmerella* sp.		KU128730	14172T	NA	NA		483	Hui,F.L.
*Starmerella* sp.	KU128718		14174T	NA	NA	424		Hui,F.L.
*Starmerella* sp.		KU128729	14174T	NA	NA		480	Hui,F.L.
*Candida* sp.	HQ658862		11864T	Plant	China	417		Li,S.L.
*Starmerella jinningensis*		HM856601	11864T	Plant	China		473	Li et al., (2013)
*Starmerella ilheusensis*	KR232375		14131T	Morning glory flowers	Brazil	409		Santos,AR.O.
*Starmerella ilheusensis*		KR232374	14131T	Morning glory flowers	Brazil		479	Santos,AR.O.
*Candida powellii*	KY102339		8795T	Insect	Costa Rica	625		Vu et al. (2016)
*Starmerella* sp. *powellii*		AF251554	8795T	Insect	Costa Rica		522	Lachance et al., (2001b)
*Starmerella floricola*	KY102086		7,289	Plant	Japan	464		Vu et al. (2016)
*Starmerella floricola*		U45710	7,289	Plant	Japan		484	Kurtzman and Robnett (1998)
*Starmerella* sp.	KM269181		14142T			426		Alimadadi,N
*Starmerella* sp.		KM269180	14142T				482	Alimadadi,N
*Candida batistae*	KY101955		8550T	Insect	Brazil	571		Vu et al. (2016)
*Starmerella batistae*		AF072843	8550T	Nesting bees	Brazil		482	Rosa,C.A.
*Starmerella caucasica*	JX112044		12650T	Flower	Azerbaijan	445		Sipiczki (2015a)
*Starmerella caucasica*		JX112043	12650T	Flower	Azerbaijan		484	Sipiczki (2015b)
*Starmerella kuoi*	HQ111058		7267T	NA	NA	953		Lachance et al., (2011)
*Starmerella bombicola*	NR121483		6009T	NA	NA	504		Lachance et al., (2011); Schoch et al., (2014)
*Starmerella bombicola*		U45705	6009T	NA	NA		482	Kurtzman and Robnett (1998)
*Starmerella riodocensis*	NR137870		10087T	NA	NA	614		Groenewald,M
*Starmerella riodocensis*		AY861674	10087T	NA	NA		524	Kurtzman and Robnett (1998)
*Starmerella lactis-condensi*	KY102179		52T	Food	United States	658		Vu et al. (2016)
*Starmerella lactis-condensi*		U45724	52T	Food	United States		485	Kurtzman and Robnett (1998)
*Starmerella camargoi*	KU710345		14130T	Tropical flower	Brazil	434		Santos,AR.O.
*Starmerella camargoi*		KR232373	14130T	Tropical flower	Brazil		427	Santos,AR.O.
*Starmerella ratchasimensis*	KY102359		10611T	Plant	Thailand	505		Vu et al. (2016)
*Starmerella floris*	KY102087		10593T	Flower	Costa Rica	485		Vu et al. (2016)
								
*Starmerella floris*		AF313353	10593T	Flower	Costa Rica		500	Lachance et al., (2001c)
*Starmerella cellae*	AY861673		10086T	Nests of the solitary bee	Brazil	940		Pimentel et al. (2005)
*Starmerella etchellsii*	AB196214		1750T			433		Suezawa et al., (2006)
*Starmerella meliponinorum*	KY105547		9117T	Insect	Brazil	702		Vu et al. (2016)
*Starmerella meliponinorum*		AF313354	9117T	Insect	Brazil		498	Lachance et al., (2001c); Teixeira et al. (2003)
*Starmerella roubikii*	MF668211		15148T	Meliponine bee	Belize	921		Ana Raquel,S.O.
*Starmerella cf. etchellsii*		AY257050		Bee	Costa Rica		493	Rosa et al. (2003)
*Starmerella khaoyaiensis*	KY102169		10839T	Plant	Thailand	457		Vu et al. (2016)
*Candida kazuoi* sp.		AB306509		Insect frass	Thailand		552	Nakase et al., (2007)
*Starmerella stellata*	AY160766		157T			432		Sipiczki (2015a)
*Starmerella stellata*		U45730	157T				481	Kurtzman and Robnett (1998)
*Starmerella davenportii*	KY102042		9069T	Insect	United Kingdom	545		Vu et al. (2016)
*Starmerella davenportii*		AJ310447	9069T	Wasp	Netherlands		488	Stratford et al., (2002)
*Starmerella bacillaris*	KY102524		9494T	Wine	Hungary	556		Vu et al. (2016)
*Starmerella bacillaris*		AY160761	9494T	Wine	Hungary		482	Sipiczki (2015b)
*Starmerella sirachaensis*	NR137646		12094T	Phylloplane of the copper pod tree	Thailand	434		Limtong et al., (2012)
*Starmerella* sp. *‘sirachaensis*		AB617909	12094T	Phylloplane of the copper pod tree	Thailand		483	Limtong et al., (2012)
*Zygoascus hellenicus*	AY447023		5839T			603		Smith et al., (2005)
*Zygoascus hellenicus*		AY447007	5839T				584	Smith et al., (2005)

The bold values under the subheading bp are base pair, and the nuclear ribosomal internal transcribed spacer (ITS) region is the formal fungal barcode and the most commonly sequenced genetic marker in mycology. Whereas, The D1/D2 domain is a 600 nucleotide domain at the 5' end of a large subunit of (26S) rDNA and most yeast species can be identified from sequence divergence of the D1/D2 domain.

This study presents a novel yeast species, the strain HSB-15^T^, isolated from the insect *Apis cerena*. The strain was found in bees frequenting flowers at the Institute of Microbial Technology, Chandigarh, India. Through morphological analysis, sugar fermentation, carbon and nitrogen source assimilation, growth profiling, and sequencing of the ITS region and D1/D2 domain of the rRNA gene, the isolate was identified as belonging to the genus *Starmerella*. It exhibited a close relationship with the *Starmerella vitae* strain, CBS 15147^T^. This discovery led to the proposal of a new taxon, *Starmerella cerana sp*. nov., for the HSB-15^T^ strain.

We also investigated the biocatalytic potential of the newly isolated yeast strain HSB-15^T^ to convert naphthyl ketones into chiral *sec*-naphthylethanols, key intermediates for the synthesis of chiral pharmaceuticals and industrial compounds. HSB-15^T^ was also explored for the production of biosurfactants. *Starmerella* sp. is well known as a producer of sophorolipid biosurfactants (Deshpande and Daniels, 1995). The produced biosurfactant was characterized and evaluated for antimicrobial activity. Our findings introduced the strain HSB-15 as a versatile microorganism serving both as a biocatalyst for naphthyl ketone reduction and a producer of the biosurfactant, sophorolipid.

## 2 Materials and methods

### 2.1 Chemicals

The chemicals and solvents used in this study were of the highest purity grade. Media components for microbial growth and culture, such as yeast carbon base (YCB) and yeast nitrogen base (YNB), were purchased from Difco (Detroit, MI). Other chemicals, such as 6′-methoxy-2′-acetonaphthone (6-MAN), 1-acetonaphthone, and 2′-hydroxy-1′-acetonaphthone, were purchased from Sigma-Aldrich. Sodium borohydride, ethyl acetate, and solvents for HPLC-grade acetonitrile, 2-propanol, and n-heptane were purchased from Rankem (India). Other media components, such as peptone, yeast extract, ultrapure agar, vitamin-free base solution, and malt extract, were obtained from Himedia (Mumbai, India).

### 2.2 Isolation of yeast species

The yeast strain used in this study was isolated from the flower-associated honey bee surface (*Apis cerana*) obtained from the IMTECH garden in Chandigarh, India, in 2014. The samples collected from the honey bee surface have been abbreviated as HSB. These samples (honey bees) were kept in sterile polyethylene bags with the associated flowers. The yeast species were aseptically isolated from the honey bee (*A*. *cerena*) surface by washing the insect with 0.8% sterile saline solution. The washed saline solution (100 µL) was plated on yeast malt agar (YMA), potato dextrose agar (PDA), and yeast peptone dextrose agar (YPDA) supplemented with 100 mg/L of chloramphenicol to reduce the bacterial growth. The pure culture of yeasts was maintained on YM agar, YEPD agar, and PDA. Furthermore, these cultures were preserved in 15% glycerol at −80 °C (Rosa et al., 2003).

### 2.3 Morphological and physiological characterization of the strain HSB-15^T^

The novel isolates were characterized using standard methods explained by Kurtzman and Fell (1998). For the biochemical characterization of yeasts, carbon source fermentation and sugar assimilation tests were performed using the Biolog YT MicroPlate (Biolog, Inc., Hayward, CA) following the manufacturer’s instructions. In order to examine the metabolic activity of the yeast strain, the Biolog YT MicroPlate was incubated for 24 h, 48 h, and 72 h. Type strains from CBS^1^ and MTCC^2^ were used for the comparison with this novel species. A sporulation test was performed on different culture media such as YMA, V8 vegetable juice agar, PDA, potato carrot agar, YCB with 0.01% ammonium sulfate, and corn meal agar at 25 °C. The assimilation of nitrogen tests were performed in test tubes. The vegetative cellular morphology and hyphae formation were observed under the scanning electron microscope.

### 2.4 Growth characteristics of the strain HSB-15^T^ at various pH values, temperatures, and media

The growth characteristics of the strain HSB-15^T^ at various pH values were measured in the autoclaved YEPD medium. Primary culture 2% (V/V) was inoculated into the secondary medium (YEPD) and incubated at 28 C for 72 h with the 200 rpm shaking condition. Cell growth was measured after a 12 h interval by measuring the optical density at 595 nm using a spectrophotometer.

To examine the growth characteristics of the strain HSB-15^T^ at different temperatures, cells were grown in a 100-mL flask containing 20 mL YEPD medium and incubated at different temperature ranges, including 4 C, 10 C, 15 C, 20 C, 25 C, 30 C, 35 C, and 40°C, for 72 h at 200 rpm, and the growth of the cells was measured as mentioned previously. Furthermore, in order to study the influence of different media on the growth of the strain HSB-15^T^, various liquid media were used including yeast malt broth (YM), yeast extract peptone dextrose (YEPD), potato dextrose broth (PDB), Luria broth (LB), nutrient broth (NB), tryptic soy broth (TYB), and Sabouraud dextrose broth (SDB). The cells were allowed to grow at 200 rpm for 72 h at 28 °C with the measurement of optical density at an interval of 12 h for 72 h.

### 2.5 DNA extraction and quantification

The yeast strains were grown on a YM agar plate at 25 °C (HiMedia, Mumbai, India) and harvested after 48 h of growth. The genomic DNA isolation was carried out using the ZR Fungal/Bacterial DNA Miniprep Kit (Zymo Research, United States). To determine the quality of the genomic DNA, electrophoresis was performed on a 0.8% agarose gel. Furthermore, the quantity and purity of DNA were determined using a NanoDrop 1000 Spectrophotometer at 260/280 (NanoDrop Technologies, Wilmington, DE, United States).

### 2.6 Amplification PCR and identification of the strain HSB-15^T^ based on D1/D2 and ITS region sequencing

Each PCR reaction was performed with a final reaction volume of 50 µL comprising 100–200 ng/μL of genomic DNA, 10 pmol of each primer, 200 µM dCTP, dGTP, dTTP, and dATP (Promega, United States), 4 mM MgCl_2_, 5 U/µL of Taq polymerase (Promega, United States), and 10 µL of 5X GO Taq Flexi buffer (Promega, United States). The primer sets NL1–NL4 were used for the PCR amplification of the D1/D2 region of the 26S rRNA gene, and the sequences of ITS (including the 5.8S rRNA gene) were amplified using the ITS1–ITS4 primer sets (Kurtzman and Robnett, 1998). The primers were obtained from Sigma-Aldrich, Bangalore, India. The amplification reaction was performed in the Proflex PCR system (Thermo Fischer Scientific, Singapore) with the following parameters: initial denaturation for 5 min at 95 °C, followed by 30 cycles of 30 s at 95 °C, 30 s at 55 °C, and 90 s at 72 °C with a final extension for 10 min at 72 °C. The amplified regions of D1/D2 and ITS were purified using the RBC HiYield Gel/PCR DNA Mini Kit (Real Biotech Corporation, India). The purified PCR product was used for the sequencing with an ABI 313 genetic analyzer (Applied Biosystems, California, United States). The complete sequences of ITS and D1/D2 regions were obtained with the primers ITS1, ITS2, ITS4, NL1, NL2A, NL3A, and NL4 (Kurtzman and Robnett, 1998). The sequences obtained were submitted in the GenBank database with the accession numbers OR470602, OR475317, and KR233472 for the ITS region and D1/D2 domain and concatenated sequences of the ITS and D1/D2 domain, respectively.

### 2.7 Classification and identification of the strain HSB-15^T^

The sequences were compared with the GenBank sequences using nBLAST and with the CBS database using pair-wise sequence alignment. The sequences were retrieved from the GenBank and aligned using CLUSTAL W (Thanh, Van Dyk, and Wingfield, 2002). The phylogenetic tree was constructed in MEGA Version 7.0 by using the neighbor-joining method and Kimura two-parameter correction with 1,000 bootstrap values (Thanh, Van Dyk, and Wingfield, 2002; Vega et al., 2012).

### 2.8 Typical experimental procedure for asymmetric ketone reduction

In order to perform biocatalytic reduction experiments, strain HSB-15^T^ cells were grown at 30°C for 48 h in shaking conditions (200°rpm); cells were further harvested by centrifugation (9,000**×** g for 10 min). The pellets were then washed twice with 0.2 M sodium phosphate buffer (pH 7.0) and re-suspended in the same buffer (100 mg/mL wet cell mass). Furthermore, 2 mM of different ketone substrates (6′-methoxy-2′-acetonaphthone, 1-acetonaphthone, and 2′-hydroxy-1′-acetonaphthone) were dissolved in 500 µL of acetone:ethanol (2:1 ratio) separately and added to a different set of reaction mixtures along with 2% glucose. All reaction mixtures were incubated at 30°C (200 rpm) in the shaker. Samples were collected (1 mL) at different time intervals (3–12 h) and centrifuged at 9,000**×** g for 10 min. The cell-free supernatants were then extracted with ethyl acetate, and the organic layer was concentrated using a rotary evaporator. The obtained dry powder was dissolved in 1 mL of methanol and subjected to reverse-phase high-performance liquid chromatography (HPLC) for monitoring the progress of the reaction.

### 2.9 Screening of the antimicrobial biosurfactant from the HSB-15 ^T^ strain

After the growth of the HSB-15 ^T^ strain, the cells were utilized for the biocatalytic reaction. However, the unused cell-free supernatant was examined for the presence of potential antimicrobial compound/s. To perform the experiment, first, ethyl acetate extraction was carried out, in which the broth medium was mixed well with an equal volume of ethyl acetate in a separating funnel and the organic layer was evaporated using a rotary evaporator (Buchi R-300). Furthermore, the dry content was dissolved in 1 mL of methanol for screening the antimicrobial properties of the ethyl acetate extract against an indicator bacterial strain, *Staphylococcus aureus* (MTCC 1430), using an agar well.

Diffusion method: Initially, *S. aureus* cells (10^7^ CFU/mL) were spread on an LB agar plate, in which a 6-mm-diameter well was created using a well borer. Thereafter, 100 µL (500 μg/mL) of the extract was added to the well, and the plate was incubated at 37°C overnight to examine the zone of inhibition.

### 2.10 Screening of the biosurfactant using oil displacement assay

The qualitative assay for the presence of the biosurfactant was carried out according to the previously reported method (Ganji et al., 2020). In brief, a Petri dish was filled with sterile distilled water, and 100 µL of edible oil was placed on top of the water. Furthermore, 10 µL of the 12 extract was added on top of the oil in the Petri dish, and the zone of oil displacement was observed visually.

### 2.11 Production of the biosurfactant from the strain HSB-15^T^

The production of the biosurfactant on a preparative scale was carried out as described previously by Haque et al. (2016). In brief, in 100 mL of Erlenmeyer flask containing 20 mL of YEPD medium, the yeast strain HSB-15^T^ was grown for 18 h and used as seed culture. Thereafter, 8 mL of the grown culture was taken and inoculated into a 2 L Erlenmeyer flask containing 400 mL of biosurfactant production media consisting of cotton seed oil (100 g/L), glucose (100 g/L), malt extract (10 g/L), and urea (1 g/L). Finally, the flask was incubated at 30 °C and 200 rpm in an orbital shaker incubator for 144 h.

### 2.12 Extraction, purification, and characterization of the biosurfactant

After 1 week of incubation of HSB-15^T^ cells in biosurfactant production media, cells were separated from the broth by centrifugation at 8,000 rpm for 20 min. Furthermore, the biosurfactant produced in the broth was extracted by the ethyl acetate extraction method. In brief, the broth of culture and ethyl acetate were mixed in a ratio of 1:1 in a separating funnel of 1L capacity and were mixed by shaking. The solvent broth mixture was left to settle down for 15 min, and two different liquid phases were created. The lower phase containing water was discarded, and the top-layer ethyl acetate part was collected separately. The solvent ethyl acetate containing biosurfactant was evaporated at 40°C by vacuum evaporation (Buchi R-300). To remove the residual hydrophobic components, the concentrated extract was washed with n-hexane (Wadekar et al., 2012). The obtained brownish oily biosurfactant was examined by thin-layer chromatography (TLC) (Sen et al., 2017). Furthermore, column chromatography was carried out to isolate the bioactive biosurfactant from the crude extract using silica gel (Haque et al., 2016). Initially, a 50 cm × 5 cm glass column was packed with 50 g of silica of 60–120 mesh size in hexane. Thereafter, an eluent containing 200 mL of chloroform/methanol was passed through the column before loading the crude biosurfactant. In a round-bottom flask, approximately 200–300 mg of crude biosurfactant was dissolved in a small volume of methanol and mixed with 3.5 gm of silica. After vigorous mixing, the solvent was evaporated at 40°C under reduced pressure. When silica was fully dried into a powder form, it was loaded into the packed column. The desired antimicrobial biosurfactant was eluted using chloroform and methanol as a mobile phase in the ratio of 98:2. The collected fractions were dried using a rotary evaporator at 40°C. The dried sample was subjected to further analysis and characterization of the biosurfactant.

### 2.13 Thin-layer chromatography of the biosurfactant

To analyze the sample in thin-layer chromatography, approximately 1 mg of the purified fraction of the biosurfactant from silica gel chromatography was dissolved in methanol and spotted on a Merck Silica Gel 60 F254 10 × 5 cm TLC plate along with a commercially available sophorolipid biosurfactant {1,4-sophorolactone 6′,6′-diacetate (Sigma-Aldrich, United States)} as a reference standard. The solvents containing chloroform/methanol/water (65:15:2) were used for the stationary phase. The separated individual compounds were visualized on the TLC plate by spraying a methanol/sulphuric acid (50:50) reagent, followed by heating at 110°C for 5 min (Dubey, Selvaraj, and Prabhune, 2014).

### 2.14 High-performance liquid chromatography of the biosurfactant

A column-purified fraction of the biosurfactant was subjected to RP-HPLC (Shimadzu, Japan) fitted with a UV detector (207 nm) and an RP-C18 column (Merck, 5 μm, 4.5 × 250 mm). The injection volume was set to 20 µL, and the solvent system, acetonitrile:water (80:20), was used as the mobile phase with a flow rate of 1 mL/min. The commercially available sophorolipid biosurfactant 1,4-sophorolactone 6′,6′-diacetate (Sigma-Aldrich, United States) was used as a reference standard. The generated chromatogram was compared with standard sophorolipid.

### 2.15 Fourier-transform infrared spectroscopy analysis

Different functional groups present in the biosurfactant were determined by Fourier-transform infrared (FT-IR) spectroscopy (Bruker optics, vortex 70). The dried biosurfactant sample was analyzed using the potassium bromide (KBr) pellet method, and spectra were collected as an average of 64 scans at a resolution of 4 cm^-1^ within the range of 3,500–500 cm-^1^.

### 2.16 Liquid chromatography–mass spectrometry of the biosurfactant

The HPLC-purified sample was used for mass and structural homologous analysis by liquid chromatography–mass spectrometry (LC–MS) (Agilent 1,290 Infinity Binary LC coupled with a 6550 iFunnel Q-TOF LC/MS system). Electrospray ionization–mass spectrometry (ESI–MS) measurements were performed in the positive ion mode for the analysis of the sample.

### 2.17 Analytical procedures

HPLC (Shimadzu) equipped with a Purospher^®^ STAR RP-18 column (Merck, 5 µm, 4.5 × 250 mm) was performed using acetonitrile: water (50:50) as the mobile phase, with a flow rate of 1 mL/min. Peaks for 6′-methoxy-2′-acetonaphthone and its corresponding alcohols were detected at 233 nm by a UV detector. In order to analyze the chirality of reduced alcohols, a Chira Select OM chiral column (Merck, 5µ, 4.5 × 250 mm) was used with heptane:2-propanol (85:15) as the mobile phase with a flow rate of 0.5 mL/min. Moreover, the synthesized alcohols by biocatalysis were further characterized by ^1^H and ^13^C NMR spectra using the multinuclear FT-NMR spectrometer model ECX-300 (JEOL, United States) with a frequency of 300 MHz for ^1^H and 100 MHz for ^13^C (see Supplementary Figures S1-S6 **)**.

## 3 Results and Discussion

### 3.1 Description of the strain HSB-15^T^

**FIGURE 1:**
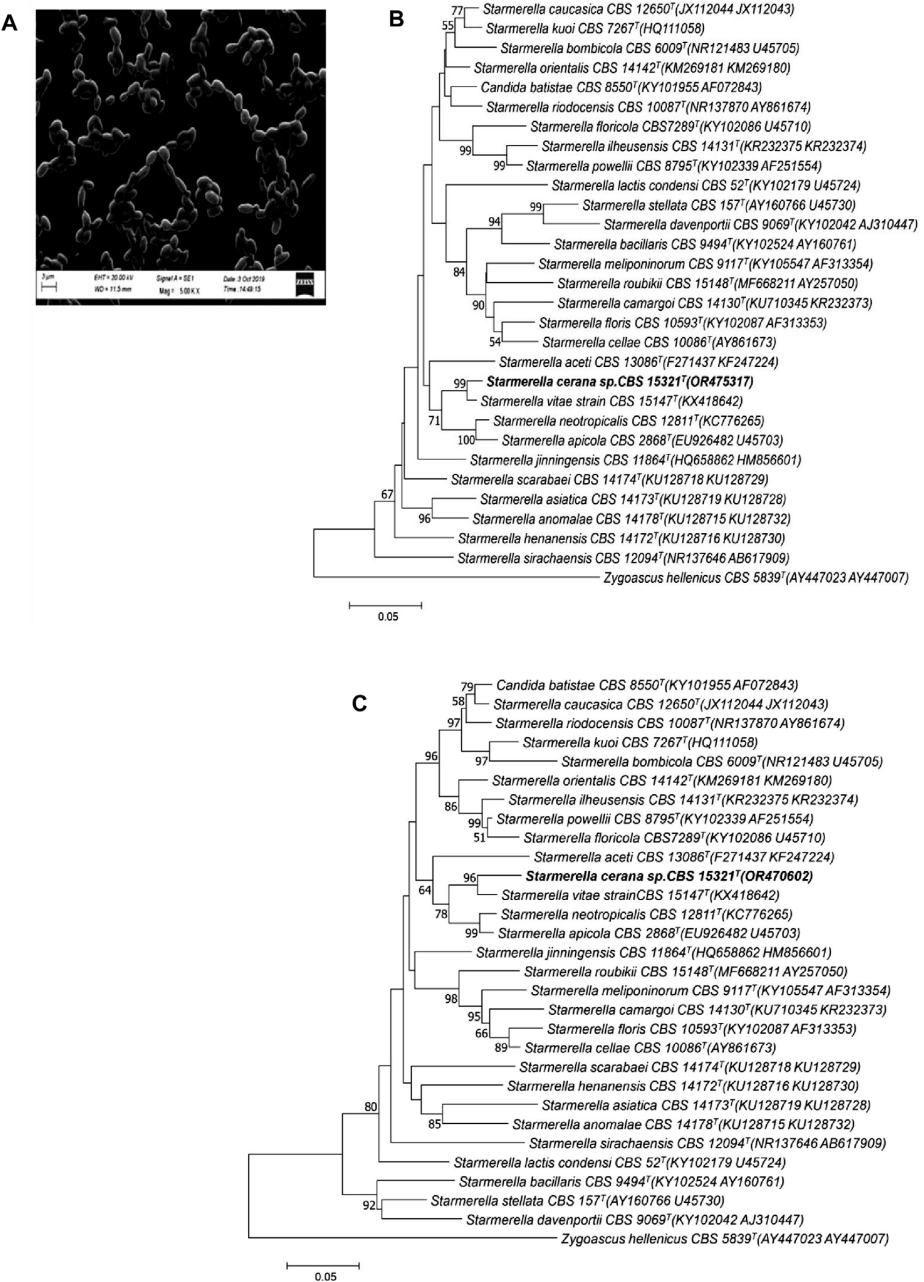
Morphological appearance and phylogenetic analysis of the strain HSB-15T (*S. cerena*). SEM image showing the morphology of the isolated strain **(A)**. Phylogenetic tree constructed on the basis of the nucleotide sequence of the D1/D2 domain of the LSU **(B)** and the ITS of the SSU **(C)** of rRNA genes, showing a close relationship with *S. vitae* CBS 15147T (KX418642) using the neighbor-joining algorithm with 1,000 bootstrap values. The distance was calculated according to the work of Kimura (1980). Bar indicates 5% variation.

Morphological analysis of the isolates was carried out by growing the strain on yeast malt agar for 5 days at 25 °C. The colonies were observed to be raised, smooth, creamy in color, and butyrous. Cells were found to have no hyphal or pseudohyphal growth under the scanning electron microscope (see Figure 1A). Cells grown on yeast malt agar (after 5 days of growth at 25 °C) were observed to be round to ovoid in shape, mainly present in chains; budding was polar, measuring 3–4 by 2–3 µm. Moreover, when the cells were cultured on corn meal agar for 21 days, they were unable to produce pseudohyphae, and the culture was devoid of any sporulation after 3–4 weeks of incubation at 25 °C.

### 3.2 Growth characteristics of the strain HSB-15^T^ at different temperatures, pH values, and media

**FIGURE 2:**
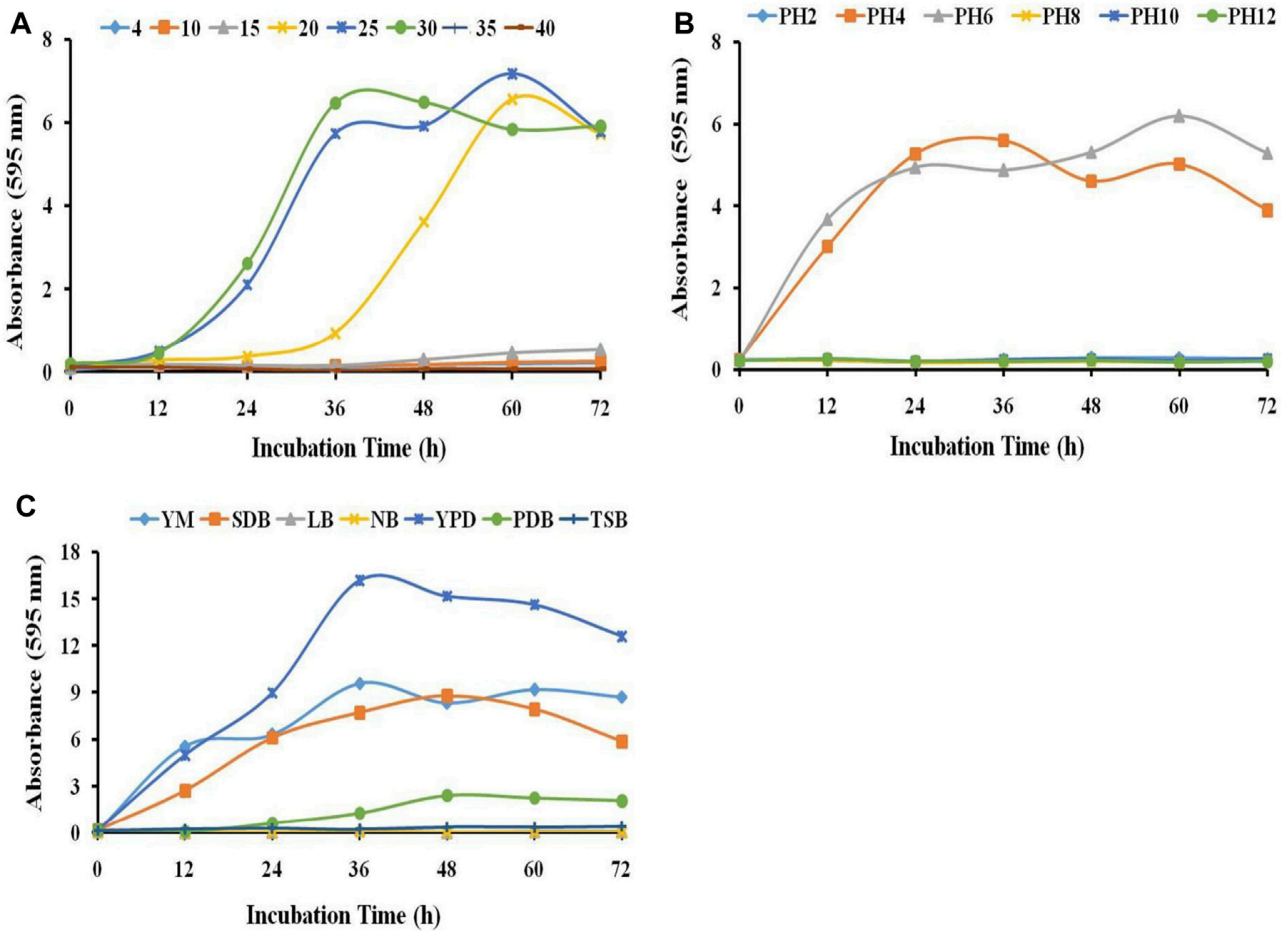
Influence of different parameters on the growth of HSB-15^T^. Effect of different temperatures **(A)**, pH values, **(B)** and growth media **(C)** on the growth of HSB-15^T^.

To study the growth characteristics of the strain HSB-15^T^ (MTCC-12380^T^) at different temperatures, the strain was inoculated in YEPD media and incubated at temperatures ranging from 4°C to 40 °C. The growth of the strain was inhibited at lower temperatures (4–15 °C) and higher temperatures (40°C). However, a significant growth characteristic of the strain was observed between 20°C and 30 °C, and the optimum growth of the strain was found to be 25 °C (see Figure 2A). The growth characteristic of the strain HSB-15^T^ was examined within the pH range of 2–12 in the YEPD medium (see Figure 2B). The strain HSB-15^T^ showed significant growth within the pH range of 4.0–6.0. However, at lower pH (below 4.0) and higher pH (above 6.0), no significant growth was detected. The growth pattern of the strain HSB-15^T^ was also examined in different liquid media including YM, SDB, LB, NB, YEPD, PDB, and TSB broth. It is evident from Figure 2C that the strain was able to grow optimally in YEPD, YM, and SDB within 12–36 h of the incubation period. However, the growth rate was retarded in PDB, LB, NB, and TSB media. All these observations indicated that the growth characteristics of the strain HSB-15^T^ showed a considerable similarity with the genus *Starmerella.*

A comparative study for the biochemical taxonomic characterization of the strain HSB-15^T^ was carried out using the Biolog system to determine the relationship with its closely related type strain *S*. *vitae* strain CBS 15147. According to phylogenetic relatedness, the strain was also compared with other related species, which showed remarkable differences with *S*. *vitae* strain CBS 15147 (type species differs in six tests), *Starmerella apicola* CBS 2868 (differs in 12 tests), *Starmerella bombicola* CBS 6009 (eight tests), and *Starmerella neotropicalis* CBS 12811 (differs in 14 tests), respectively (see Table 2). Tests on the fermentation and assimilation of sugars confirmed that the strain HSB-15^T^ was capable of fermenting D-glucose but was unable to ferment maltose. Furthermore, the sugar substrates trehalose and raffinose were also fermented by the novel strain; however, the same substrates were not utilized by other related-type strains. Furthermore, the sugar substrate L-sorbose was not utilized by the strain HSB-15^T^, whereas *S*. *vitae* strain CBS 15147, *S*. *apicola* CBS 2868, *S*. *bombicola* CBS 6009, and *S*. *neotropicalis* CBS 12811 were capable of utilizing the substrate. The strain HSB-15^T^ was shown to be weakly positive for assimilation of D-xylose and ribitol, which was not assimilated by its type strains *Starmerella cf. bombi* CBS 15147, *S. apicola* CBS 2868, *S. bombicola* CBS 6009, and *S. neotropicalis* CBS 12811. Assimilation of trehalose was positive for the strain HSB-15^T^, whereas other related-type strains were unable to assimilate the same carbon sources. The study of various temperatures showed that the strain HSB-15^T^ was capable of growing at temperatures ranging from 20°C to 30°C, although the strain HSB-15^T^ and other related species were unable to grow at higher temperatures (37°C). However, the strain *S. apicola* CBS 2868 can grow up to 37°C. The growth of HSB-15^T^ in 1% yeast extract and 2% agar medium containing 10% and 16% NaCl was found to be negative, but the *S. vitae* strain CBS 15147 was shown to be positive in the medium with 10% NaCl. In the presence of 60% glucose, the growth of the HSB-15^T^ strain was found to be negative. However, other type strains, such as *S. apicola* CBS 2868 and *S. bombicola* CBS 6009, were shown to be positive under similar conditions. Acid production was positive for the strain HSB-15^T^, whereas *S. apicola* CBS 2868, *S. bombicola* CBS 6009, and *S. neotropicalis* CBS 12811 were observed to be non-acidogenic. These tests indicated remarkable differences in the isolated strain HSB-15^T^ when compared to other described related strains.

**TABLE 2:** Biochemical properties of *S. cerena* HSB-15^T^.

Biochemical properties	A	B*	C*	D*	E^†^
Fermentation of					
Maltose	-	NR	-	-	-
α-D-glucose	+	w, d	+	+	d
Sucrose	+	NR	w, d	+	d
Trehalose	+	NR	-	NR	-
Raffinose	wp	NR	-	wp	-
**Assimilation of**					
L-sorbose	-	+	+	d	d, w
D-glucosamine	-	NR	-	-	-
D-ribose	-	-	d	-, d, w	-
D-xylose	wp	-	-	-	-
L-arabinose	-	-	-	-	-
Methyl-a-d-glucoside	-	NR	-	-	-
Cellobiose	-	-	-	-	-
Salicin	-	-	-	-	-
Arbutin	-	NR	-	-	-
Raffinose	+	+	+	-, d, w	-
Ribitol	wp	-	-	-	-
Xylitol	-	-	-	-	-
L-arabinitol	-	NR	-	-	w
Galactitol	-	-	-	-	d, w
Ethanol	ND	-	+	+	-
Trehalose	+	-	-	-	-
**Growth at**					
25°C	+	+	+	+	+
30°C	+	NR	+	+	+
35°C	-	NR	+	-	-
37°C	-	-	+	-	-
10% NaCl	-	+	NR	NR	-
16% NaCl	-	NR	NR	NR	v
50% Glucose	+	+	+	+	-
60% Glucose	-	NR	+	+	NR
Acid production from glucose	+	NR	-	-	-
On 0.1% cyclohexamide	ND	NR	NR	-	-
vitamin free	ND	NR	-	-	-

^a^ , positive; -, negative; wp, weak positive; d, delayed; dw, delayed weak; ND, not determined; NR, not reported. The four strains of *Starmerella* genus were used for the physiological characterization test. A, *S. cerena* CBS 15321, HSB-15 (MTCC, 12380) sp. nov.; B, *S*. *vitae* CBS 15147; C, *S. apicola* CBS 2868; D, *S. bombicola* CBS 6009; E, *S. neotropicalis* CBS 12811. The reference strain data were taken from the CBS yeast database and the work of Daniel et al. (2013).

^b^ = Data from the CBS yeast database.

^c^ = Data from the work of Daniel et al. (2013).

### 3.3 Phylogeny of the new *Starmerella* species

To determine the phylogenetic position of *S*. *cerena* (HSB-15 T strain), a phylogenetic tree was constructed by the pair-wise alignment of the ITS region (465 bp), which showed 3.7% sequence divergence, whereas the D1/D2 region (595 bp) showed 2.5% sequence divergence from *S. vitae* strain CBS 15147 T. According to Kurtzman and Robnett (1998), established rules for classifying ascomycetous yeast species using nucleotide divergences state that strains that show more than six nucleotide sequence changes (equal to 1% sequence divergent) in the D1/D2 domain may be considered as the limit for the discrimination of two species. A taxonomic differentiation threshold was subsequently established by Vu et al. (2016), who stated that a strain should be classified as a distinct species in comparison to its close neighbors if it exhibits a similarity of less than 98.31% (for Ascomycota) or 98.61% (for Basidiomycota) within the ITS region. The suggested criterion for distinguishing species is modified to less than 99.41% similarity (for Ascomycota) or 99.51% similarity (for Basidiomycota) in the case of D1/D2 domain analysis. Thus, based on the sequence divergence of the ITS region and D1/D2 domain, the HSB-15^T^ strain is considered a new species. Moreover, when the analysis was carried out with the ITS and D1/D2 domain of the novel strain HSB-15 T and closely related *Starmerella*, clade species were used to construct a neighbor-joining phylogenetic tree. In the phylogenetic analysis of strain HSB-15^T^, it was found to be closely related to species *S. vitae* strain CBS 15147 with strong 96 and 97 bootstrap values for the ITS and D1/D2 domain, respectively (see Figure 1B). The comparative analysis of multiple sequence alignments has revealed distinct genetic variations in the strain HSB-15 T when compared to closely related types: from the *S. vitae* strain by more than 4% nucleotide divergence (13 substitutions and four gaps), from *S. apicola* by 7% (28 substitutions and six gaps), and from *S. neotropicalis* by 8% (25 substitutions and 12 gaps), all within the ITS region, and from the *S. vitae* strain by 2% nucleotide divergence (nine substitutions and three gaps), from *S. apicola* by 7% (25 substitutions and eight gaps), and from *S. neotropicalis* by 6% (27 substitutions and four gaps) based on the D1/D2 domain. *Zygoascus hellenicus* CBS 5839 T was used as an out group in this study. Thus, it was proved that the strain belongs to a distinct taxon of the clade *Starmerella* and may be designated as a different species named *Starmeralla cerana.*

### 3.4 Reduction of 6′-methoxy-2′-acetonaphthone to *S*-1-(6-methoxy-2-naphthyl) ethanol in a different time course

**FIGURE 3:**
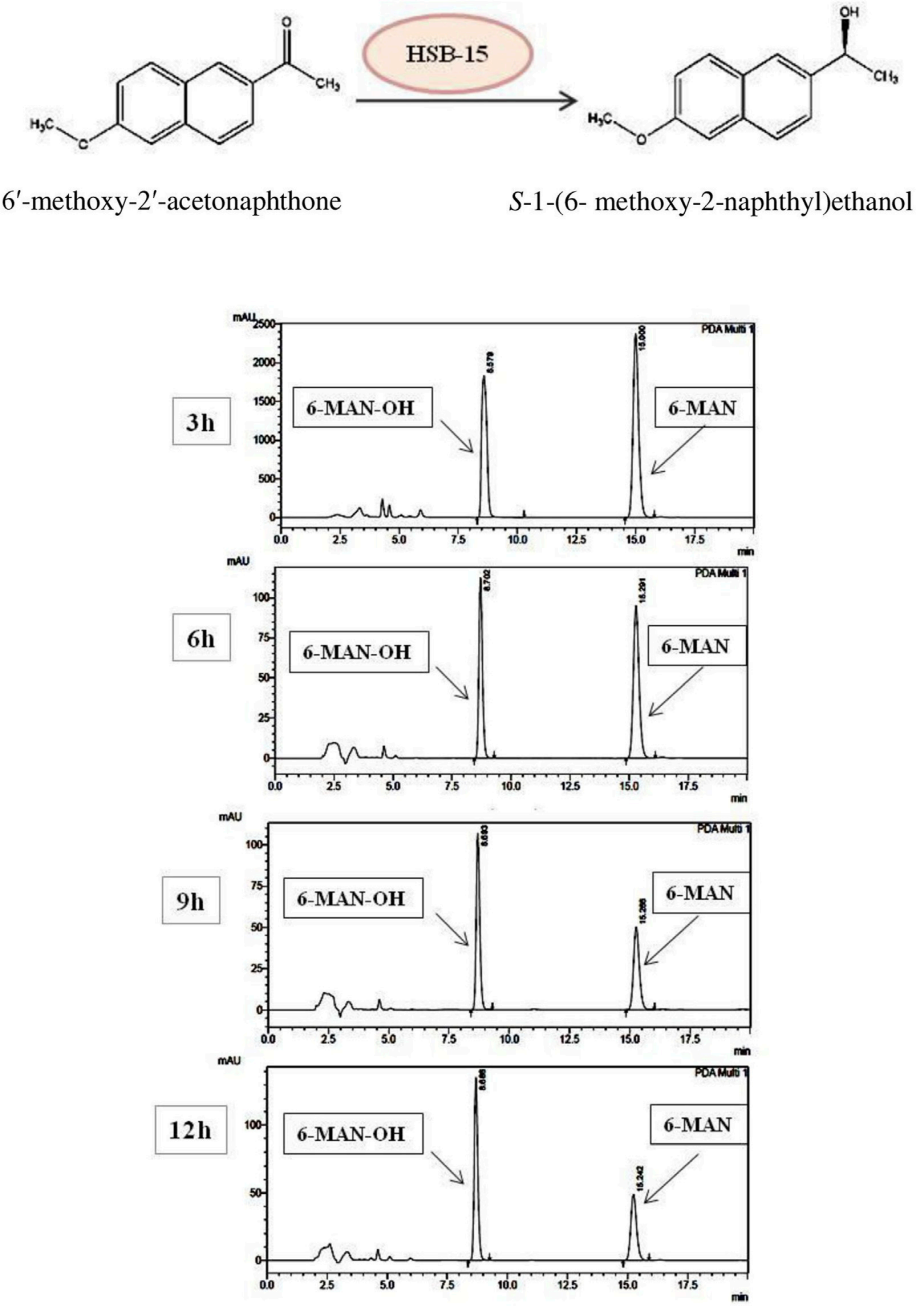
Schematic representation of the biocatalytic reduction carried out by HSB-15^T^ (*S. cerana*) (A). HPLC chromatogram shows the time course of the biocatalytic reduction of 6′-methoxy-2′-acetonaphthone to (*S*)-1-(6-methoxy-2-naphthyl) ethanol by HSB-15^T^ (*S. cerana*).

Conversion of 1-(6-methoxy-2-naphthyl) ethanol from its prochiral ketone by cells of the HSB-15^T^ strain as whole-cell biocatalysis was carried out, and the progress of the reaction at different time intervals was monitored by TLC and HPLC equipped with the RP-C 18 Column. The time course of biocatalysis is depicted in Figure 3 by the strain HSB-15^T^ showing selective conversion of 1-(6-methoxy-2-naphthyl) ethanol from the prochiral substrate in 3 h–12 h (Table 3) with the highest percentage of conversion (63.3%), within 12 h (Table 4). Since the synthesis of enantiopure drugs has been compelled by different regulatory agencies (United States Food and Drug Administration, European Medicines Agency, *etc.*), this study provides an exploration of biocatalysts for the synthesis of the industrially relevant compound *S*-1-(6-methoxy-2-naphthyl) ethanol (6-MAN-OH), which is a key intermediate of the anti-inflammatory drug naproxen, which is a racemic drug. Furthermore, in order to expand the window of the substrates, the novel strain HSB-15^T^ was tested for different naphthyl ketones. Our biocatalytic study reveals the substrate acceptance of the strain HSB-15^T^. The strain was capable of reducing 6′-methoxy-2′-acetonaphthone, 1-acetonaphthone, and 2′-hydroxy-1′-acetonaphthone with the highest yields (63.3%, 72.6%, and 23.5%, respectively) in 12 h.

**TABLE 3:** Synthesis of 6′-methoxy-2′-acetonaphthone to *S*-1-(6-methoxy-2-naphthyl) ethanol by whole-cell biocatalysis using the strain HSB-15^T^.

Time (h)	Conversion (%)
3	42.5 ± 3.1
6	54.2 ± 2.1
9	56.9 ± 1.6
12	63.3 ± 1.6

**TABLE 4:** Reduction of different prochiral naphthyl ketone derivatives to enantiopure S-specific alcohols in 12 h by whole-cell biocatalysis using the strain HSB-15^T^.

Substrates	Product alcohols	Conversion (%)
2′-Hydroxy-1′-acetonaphthone	(*S*)-1-(2-hydroxy-1-naphthyl)ethanol	23.5 ± 0.7
6′-Methoxy-2′-acetonaphthone	(S)-1-(6-methoxy-2-naphthyl)ethanol	63.3 ± 1.3
1-Acetonaphthone	(S)-(−)-1-(1-naphthyl)ethanol	72.6 ± 0.9

Numerous documented studies highlight the effective utilization of yeast cells as whole-cell biocatalysts for reducing naphthyl ketones to yield naphthyl alcohols. These reports underscore the versatility and efficiency of yeast cells in this bioconversion process. For instance, in a notable study led by Roy et al. (2003), *Geotrichum candidum* and *Candida parapsilosis* were found to efficiently catalyze the reduction of 1-acetonephthone to S (−)-1-(1‘-naphthyl) ethanol. The conversion rates reached 84% and 43%, respectively, within 24 h (Roy et al., 2003). This biocatalyzed product, *S* (−)-1-(1‘-naphthyl) ethanol, plays a crucial role as an intermediate in mevinic acid analog synthesis, serving as a promising inhibitor of 3-hydroxy methyl glutaryl coenzyme A reductase (HMGR), commonly known as statins, used to treat hyperlipidemia. Subsequently, Bhattacharyya and Banerjee (2007) conducted a study exploring the production of the carbonyl reductase enzyme responsible for the bioconversion in *G. candidum*. Following the optimization of various media components and physicochemical parameters, the conversion of 1-acetonephthone to its corresponding product increased to approximately 93% (Bhattacharyya and Banerjee, 2007). In a separate study, *Rhodotorula glutinis* yeast cells acted as whole-cell biocatalysts for producing *S* (−)-1-(1‘-naphthyl) ethanol, achieving a conversion rate of 100% after optimization (Kurbanog et al., 2008). Zilbeyaz et al. (2016) reported a preparative-scale conversion of 1-acetonaphthone to (*S*)-(−)-1-(1’-naphthyl) ethanol by the fungus *Alternaria alternata*, yielding up to 82% in 48 h (Zilbeyaz, Kurbanoglu, and Kilic, 2016). In a prior study, multiple isolated yeast strains were examined for reducing 6′-methoxy-2′-acetonaphthone to (*S*)-1-(6-methoxy-2-naphthyl) ethanol. The yeast strain CHF-15P, identified as *Rhodotorula kratochvilovae*, exhibited the highest conversion potential. It was proficient in reducing five different naphthyl ketone substrates to their respective naphthyl alcohols, with exceptional conversions achieved in substrates such as 6′-methoxy-2′-acetonaphthone and 4′-fluoro-1′-acetonaphthone, surpassing 95% and 96%, respectively (Verma et al., 2021). Subsequently, in 2023, Preeti et al. reported the synthesis of (*S*)-(−)-1-(1′-naphthyl) ethanol employing *Pichia kudriavzevii* cells, yielding a conversion of 75% (Preeti et al., 2022).

Although the majority of studies have reported single substrates for bioconversion (except the work of Verma et al. (2021)), the novel strain *S. cerena* (HSB-15^T^ strain) exhibits promising capabilities by effectively reducing three distinct substrates, yielding pivotal drug intermediates. Notably, the conversion time is also shorter compared to that reported in many of these studies (Verma et al., 2021). Nonetheless, while most of these studies demonstrated high substrate conversion using distinct yeast cells, it is essential to note that the meticulous optimization process encompassing a spectrum of physicochemical parameters, which unlocks their ultimate conversion potential, is not addressed in the scope of this particular study.

### 3.5 Production of sophorolipid biosurfactant from the strain HSB-15 and examination of its antimicrobial properties

**FIGURE 4:**
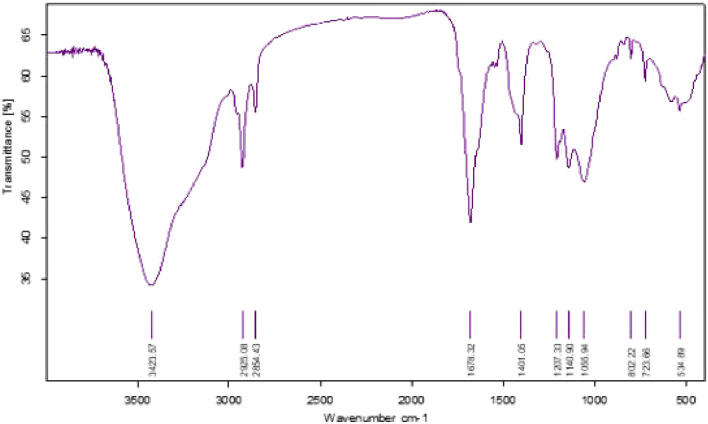
FT-IR spectra of the biosurfactant produced by the novel yeast strain HSB-15^T^.

**FIGURE 5:**
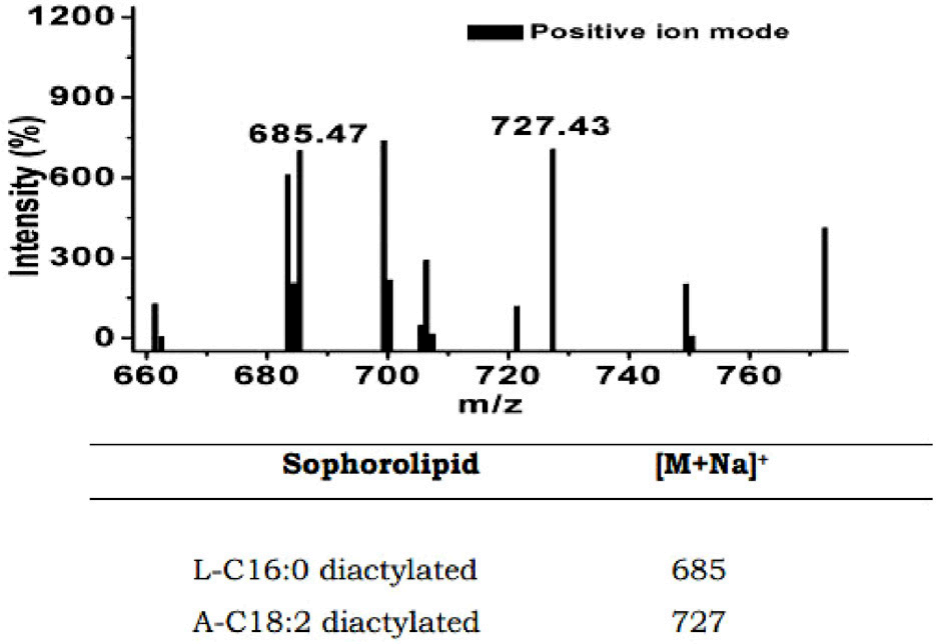
LC–MS spectra showing the m/z values of 685 and 727 for putative sophorolipid biosurfactant produced by HSB-15^T^ cells.

In this study, we found that the HSB-15^T^ strain possesses the capability to produce a biosurfactant. This biosurfactant was subsequently examined for potential biological activities. Notably, due to the close genetic resemblance of the strain HSB-15^T^ to the *Starmerella* genus, it was initially hypothesized that a similar type of biosurfactant might be produced. Nevertheless, our findings demonstrated that the biosurfactant produced by HSB-15^T^ exhibited a remarkable ability to inhibit the growth of microorganisms. The biosurfactant was isolated by the ethyl acetate extraction method and partially purified by LH-20 gel column chromatography. Final purification was performed by RP-HPLC (Supplementary Figure S8). The biosurfactant produced was subjected to various characterization techniques, and the results indicated its resemblance to the biosurfactant produced by *Starmerella* sp. Through FT-IR analysis, a comparison with the existing literature revealed a similarity to sophorolipid (see Figure 4). This initial identification was subsequently corroborated by LC–MS analysis (detailed data are presented later in the manuscript), as shown in Figure 5. The evaluation of the antibacterial activity of a purified sophorolipid biosurfactant against *S. aureus* was carried out using the agar-well diffusion method. As shown in Supplementary Figure S8, 100 µL of the HPLC-purified sample (500 μg/mL) was added to the LB agar plate containing *S. aureus* cells. After incubation of the plate at 37°C overnight, a clear zone of inhibition was observed.

Notably, the diameter of this inhibitory zone measured approximately 20 mm. In the literature, the antibacterial activity of sophorolipid against *S. aureus* has been reported by several research groups. For instance, Ma et al. (2022) reported that the MIC value of sophorolipid was determined to be 1.5625 mg/mL against *S. aureus* (Ma et al., 2022). Similarly, da Fontoura et al. (2020) reported a distinct MIC value of 500 mg/mL for sophorolipid in combating the human pathogen *S. aureus* (da Fontoura et al., 2020). Chen and Zhifei (2020) reported the antibacterial activity of sophorolipid against *S. aureus.* In the agar-well diffusion assay, a 9-mm-diameter clear zone of inhibition was observed with 50 µL of sophorolipid (2.5 mg/mL). Furthermore, they evaluated the MIC value, which was determined to be 32 μg/mL through a double broth dilution method (Chen and Zhifei, 2020). In a separate study, while evaluating a comparative study of sophorolipid-capped gold nanoparticles with free sophorolipid, Shikha et al. (2020) reported a zone of inhibition of 15 mm diameter by applying 100 µL of sophorolipid with a concentration of 400 μg/mL (Shikha, Chaudhuri, and Bhattacharyya, 2020).

It is worth mentioning that the variation in MIC values discerned across diverse research groups could potentially be attributed to the inherent structural complexity of sophorolipid. Factors such as differing chain lengths, the presence of saturated or unsaturated fatty acid chain, acetylation patterns, and the homogeneity of sophorolipid compounds collectively contribute to the observed variability in MIC values.

### 3.6 Ecological aspect of the *Starmerella* clade

The strain isolated from the honey bee (*A. cerena*) surface, collected from the IMTECH garden, is a flower-associated strain. The majority of yeasts from the *Starmerella* clade are associated with insect vectors such as bees and the substrates that these insects often visit. Flowers, fruits, bees, honey, honey bread, etc., are common sites from which many of the species of the genus *Starmerella* were isolated (Gilliam, 1979; Masneuf-Pomarede et al., 2015; Alimadadi et al., 2016). The presence of yeasts of the *Starmerella* clade on the surface of bees or at their associated sites is the basis of a belief that they exist mutually in the beneficial relationship between bees and different species from the *Starmerella* clade (Rosa et al., 2003; Oliveira et al., 2014). Since flowers provide suitable microenvironments for the growth of various yeasts, the floral parts, especially flower nectar (with a high-sugar-concentration microenvironment), are one of the important habitats of the members of the genus *Starmerella*. They are generally osmotolerant yeasts due to their survival in high-sugar-concentration habitats (Sipiczki, 2015a; Amoikon et al., 2018). The first isolated species from the *Starmerella* clade, i.e., *S. bombicola* (well known for sophorolipid production), was discovered in the honey of bumblebees in Canada (Graeve et al., 2018). Thereafter, various species from the same clade were isolated from similar habitats (flowers, insects, or the substrates where these insects visited). For example, *Candida riodocensis* and *Candida cellae* from the *Starmerella* clade were isolated in solitary bees in Brazil (Pimentel et al., 2005). *Torulopsis magnoliae* was associated with pollen that was stored in comb cells of bee bread of *Apis mellifera* (Saksinchai et al., 2012). The yeast strain *Starmerella bacillaris* was found to be associated with grapefruit and wine environments (Masneuf-Pomarede et al., 2015). Other species from the same clade were isolated from flower sources; for example, *Starmerella orientalis* was isolated in Iran (Alimadadi et al., 2016), *Starmerella jinningensis* was isolated in China (Li et al., 2013), and *Starmerella syriaca* was isolated in Syria (Sipiczki, 2015a). The frequent distribution of *Starmerella* clade species in specific ecological niches provides their physiological adaptation in high-sugar-concentration environments. Moreover, the association with bees is part of a mutual relationship that helps in developing plant–animal interactions.

### 3.7 Characterization of the sophorolipid biosurfactant

In several literature reports, it is well described that yeasts from the genus *Starmerella* are known for the production of a glycolipid biosurfactant called sophorolipid (Jezierska, Claus, and Van Bogaert, 2018). Since, in the present study, the strain HSB-15T was identified as being closely related to the genus *Starmerella*, it was hypothesized that the novel yeast HSB-15^T^ could be a sophorolipid-producing strain (C. P. Kurtzman et al., 2010). Therefore, when cells were grown for biocatalytic experiments, the broth after the separation of the cells was utilized for this purpose. To examine the presence of biosurfactant in the broth, an oil displacement assay was carried out. It is evident from Supplementary Figure S7A that oil in the Petri dish was spread after the addition of a 10 µL sample, indicating the presence of surfactant in the sample. Furthermore, the characterization of the biosurfactant was initially carried out using thin-layer chromatography and high-performance liquid chromatography. A comparative study of the produced biosurfactant by HSB-15^T^ with commercially available sophorolipid biosurfactant (1,4-sophorolactone 6′,6′-diacetate) using TLC showed a similar banding pattern as the reference standard. Moreover, the TLC result also revealed the presence of both acidic and lactonic forms of sophorolipid biosurfactant (Supplementary Figure S7B). Furthermore, the isolated biosurfactant sample was also subjected to reverse-phase HPLC along with the standard sophorolipid. The 21 chromatograms of the HPLC clearly indicated the presence of sophorolipid in the sample, as the retention time of the biosurfactant was found to be similar to the reference standard.

### 3.8 FT-IR analysis of the biosurfactant

After the purification of biosurfactant using different chromatographic steps, the purified biosurfactant sample was subjected to FT-IR analysis. Thereafter, the elucidation of the functional groups attached to the biosurfactant was carried out by the FT-IR spectrum. The absorption spectra of the biosurfactant shown in Figure 4 revealed the identity of the compound. A broad absorption band present at 3,423.57 cm^-1^ corresponds to the O–H stretch, indicating the presence of the sugar moiety in the biosurfactant. Another two peaks at 2925.08 cm^-1^ and 2854.43 cm^-1^ correspond to the C–H band, representing the aliphatic hydrocarbon tail of the biosurfactant. A peak of C–H bending is observed at an absorption band of 1,678.32 cm^-1^. Since the carboxylic acid group is present in the acidic sophorolipid, the presence of O–H bending is indicated by the absorption at 1,401.05 cm^-1^. The strong peaks in the range of 1,000–1,400 cm^-1^ contribute to C–O stretching, indicating a lactone, ester, or acid group in biosurfactant samples. A peak at 802.22 cm^-1^ shows CH bending and its distribution at 1 and 3 positions. The distribution of unsaturation in the long hydrocarbon tail can also be seen as C=C bending by an absorption band at 723.66 cm^-1^. The spectra obtained from FT-IR analysis clearly indicate the chemical constituents of the purified compound, confirming it as a glycolipid biosurfactant. A comparative study with the spectrum available in previous studies (Akbari et al., 2020; Ganji et al., 2020) reveals the purified biosurfactant from HSB-15^T^ to be a sophorolipid biosurfactant.

### 3.9 Mass analysis of the sophorolipid biosurfactant

The mass analysis of the sophorolipid biosurfactant was carried out by LC–MS in the positive ion mode. The analysis of the LC–MS result shown in Figure 5 reveals the molecular weight and presence of structural homologs of the sophorolipid biosurfactant produced by HSB-15^T^. The characteristic ion peak at m/z of 685 [(M + Na) +] suggests the presence of a lactonic diacetylated form of sophorolipid containing a C16:0 fatty acid tail. The presence of another prominent peak with an m/z of 727 corresponds to diacetylated acidic sophorolipid with a linoleic acid tail (C18:2). The obtained results were compared with previously reported articles (Joshi-Navare, Khanvilkar, and Prabhune, 2013; Elshafie et al., 2015) confirming the biosurfactant to be the sophorolipid of two different homologs.

## 4 Conclusion

In the present study, the exploration of biodiversity for novel yeasts was carried out. A novel yeast species designated as HSB-15 from the surface of the honey bee was isolated and identified by amplifying the ITS and D1/D2 domain of the large subunit (LSU) of the rRNA gene. The construction of the phylogenetic tree suggests that the new species belongs to the *Starmerella* clade and is closely related to the *S. vitae* strain CBS 15147. Therefore, the name of the novel yeast was suggested as *Starmerella cerana.* Another group working on yeast isolated from bees reported Starmerella to be the most common yeast species in honey bee-stored bee bread. Yeast obtained from bees’ honey stomachs along with pollen pellets collected from bee legs had *Metschnikowia* species in abundance (Detry et al., 2020). Furthermore, various physicochemical parameters such as pH, temperature, and different media for optimum growth were examined and found to be 4–6 for pH and 20°C–30°C for temperature, and the best media for strain HSB-15^T^ growth were YEPD, YM, and SDB. Furthermore, biochemical characterization of the strain HSB-15^T^ was carried out and compared with its type strain and type species, i.e., the *S*. *vitae* strain CBS 15147*, S. apicola* CBS 2868, *S. bombicola* CBS 6009, and *S. neotropicalis* CBS 12811, respectively. Furthermore, to explore the application potential of the strain HSB-15^T^ *,* the biocatalysis for the reduction of naphthyl ketone derivatives (the key intermediates of various pharmaceuticals and industry-relevant compounds) was examined. The yeast strain HSB-15^T^ showed significant potential for reducing these prochiral ketones to the enantiopure *S*-specific alcohols. The strain demonstrated its ability to efficiently reduce 6′-methoxy-2′-acetonaphthone, 1-acetonaphthone, and 2′-hydroxy-1′-acetonaphthone, yielding remarkable results (63.3%, 72.6%, and 23.5%, respectively) within a 12-h timeframe. Javidnia *et al.* showed that *R. glutinis*, an aerobic yeast, could achieve substantial reductions in nearly all prochiral ketones, exhibiting both high conversion rates and exceptional optical purity, and the conversion efficiency was approximately 38–45% (Javidnia et al., 2016). However, *S. bombicola* is used for the production of sophorolipid, with an easily attainable yield of approximately 42.81 g/L (Alfian et al., 2022).

The strain HSB-15 could be used for the production of a biosurfactant known as sophorolipid. This biosurfactant was also evaluated for its antimicrobial and surface tension-lowering properties. The strain provided a moderate amount of sophorolipid (15 g/L) production as the media were not specially optimized for higher production. An increased amount of sophorolipid can be achieved by medium optimization and by using oil as one of the sources of lipid in the media (Elshafie et al., 2015).

Hence, the novel stain can be used for the biocatalysis of pharmaceutical drugs along with the production of sophorolipid as a biosurfactant.

## Data Availability

The original contributions presented in the study are included in the article/Supplementary Material; further inquiries can be directed to the corresponding author.

## Footnotes

1 CBS-https://wi.knaw.nl

2 MTCC-https://mtccindia.res.in/catalog

## Abbreviations

ITS, internal transcribed spacer; SEM, scanning electron microscopy; LSU, large subunit; SSU, small subunit; HPLC, high-performance liquid chromatography; and NMR, nuclear magnetic resonance.
