# Supplementary material for: Harnessing dual applications of a novel ascomycetes yeast, Starmerella cerana sp. nov., as a biocatalyst for stereoselective ketone reduction and biosurfactant production
Source: Front Bioeng Biotechnol. 2023 Oct 24;11:1264826. doi: 10.3389/fbioe.2023.1264826 (PMC10628682; doi:10.3389/fbioe.2023.1264826)
Supplement: Supplementary file 1 [file DataSheet1.docx]

**Supplementary Information**

**Spectral characterization of biocatalyzed products (naphthyl alcohols)**

The biocatalyzed alcohols were characterized by NMR spectroscopy. For that, ^1^H and ^13^C NMR spectra were recorded in CDCl_3_ solution using multinuclear FT NMR Spectrometer model ECX-300 (JEOL, USA) with frequency 300 MHz for ^1^H and 100 MHz for ^13^C.

**NMR spectra of (*S*)-1-(2-Hydroxy-1-naphthyl)ethanol**

**^1^H NMR** (300 MHz, CDCl_3_, δ in ppm)**:**8.10-8.07(m,3H, 1’-,4’-,8’-H),7.89-7.86(m,1H, 5’H),3.49-3.48 (m,1H, 1-H), 2.87-2.86 (d,3H,CH_3_), 1.61, 0.004. **^13^C NMR** (100 MHz, CDCl_3_, δ in ppm)**:**204.6(C-2’), 164.13 (C-6’), 137.56 (C 4a), 132.01 (C-8’), 129.66 (C-1’), 128.67 (C-4’), 124.45 (C-3’), 123.87 (C-8a),120.02 (C-7’), 114.97 (C-5’), 32.70(CH_3_).

**NMR spectra of (*S*)-1-(1-naphthyl)ethanol**

**^1^H NMR** (300 MHz, CDCl_3_, δ in ppm): 8.10-7.79 (m,3H, 4’-,5’-,8’-H), 7.53-7.48 (m,4H, 2’- 3’-,6’-7’-H), 5.68-5.66 (m,1H, 1-H), 2.16,1.96, 1.95, 1.68-1.66 (d,3H,CH_3_), 1.58, 0.0051.**^13^C NMR** (100 MHz, CDCl_3_, δ in ppm): 141.43 (C-2’), 133.91(C-4a’), 128.98 (C-8a’), 128.04(C-5’), 126.12 (C-7’), 125.62 (C-6’), 125.62 (C-3’), 123.26(C-2’), 122.08 (C-8’), 67.23 (C-1), 24.44 (CH_3_).

**NMR spectra of1-(6-methoxy-2-naphthyl)ethanol**

**^1^H NMR** (300 MHz, CDCl_3_, δ in ppm)**:**7.74-7.70 (m,3H, 1’-,4’-,8’-H), 7.49- 7.45 (m,1H, 5’H), 7.15-7.13(m,2H, 3’-,7’-H), 5.04- 5.02 (m,1H, 1-H), 3.91 (s,3H,OCH_3_)., 1.88, 1.87, 1.58, 1.56-1.56 (d,3H,CH_3_).**^13^C NMR** (100 MHz, CDCl_3_, δ in ppm)**:** 157.74 (C-6’), 141.00 (C-2’), 134.14 (C 4a), 129.49 (C-8’), 128.83 (C-1’), 127.25 (C-4’), 124.45 (C-3’), 123.86 (C-8a),119.05 (C-7’), 105.77 (C-5’), 70.62(C-1), 55.39 (OCH_3_),25.68(CH_3_).


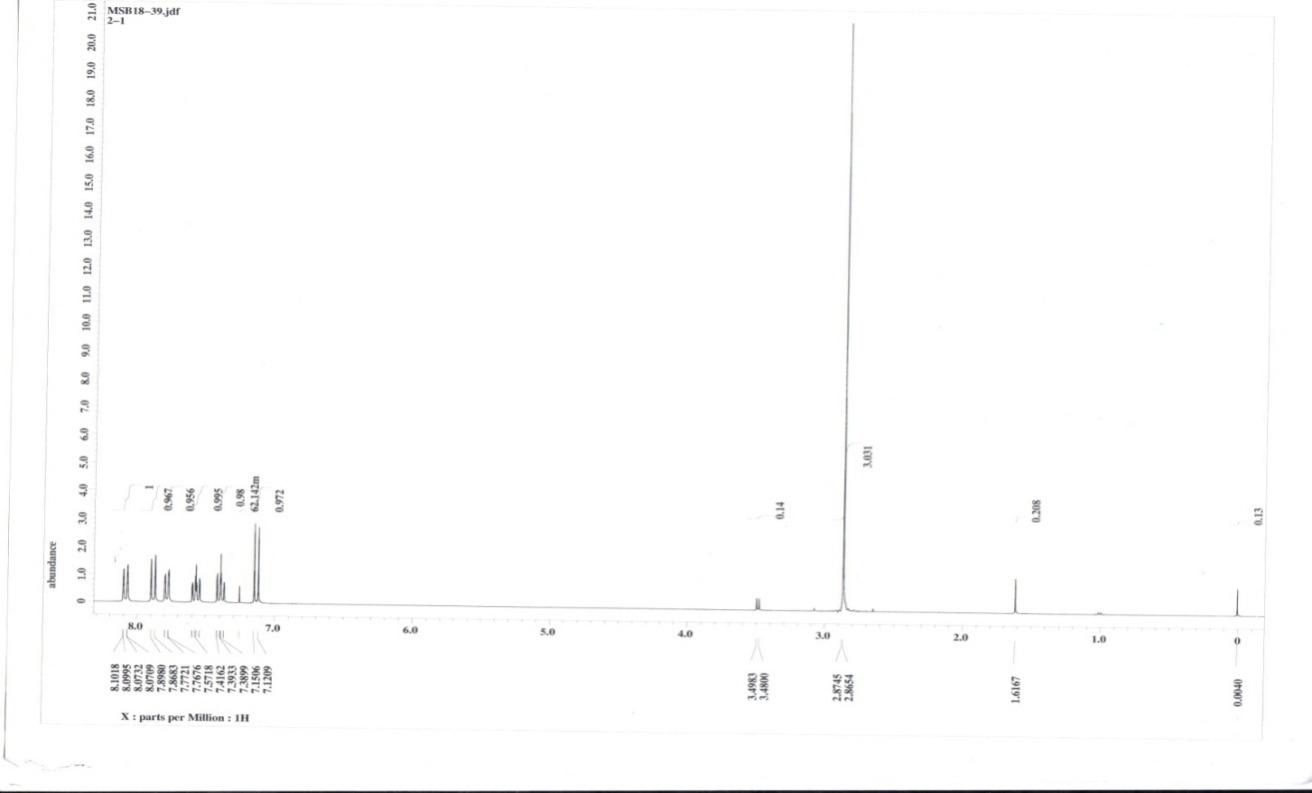


**Supplementary figure1** ^1^H NMR spectra of (*S*)-1-(2-Hydroxy-1-naphthyl) ethanol


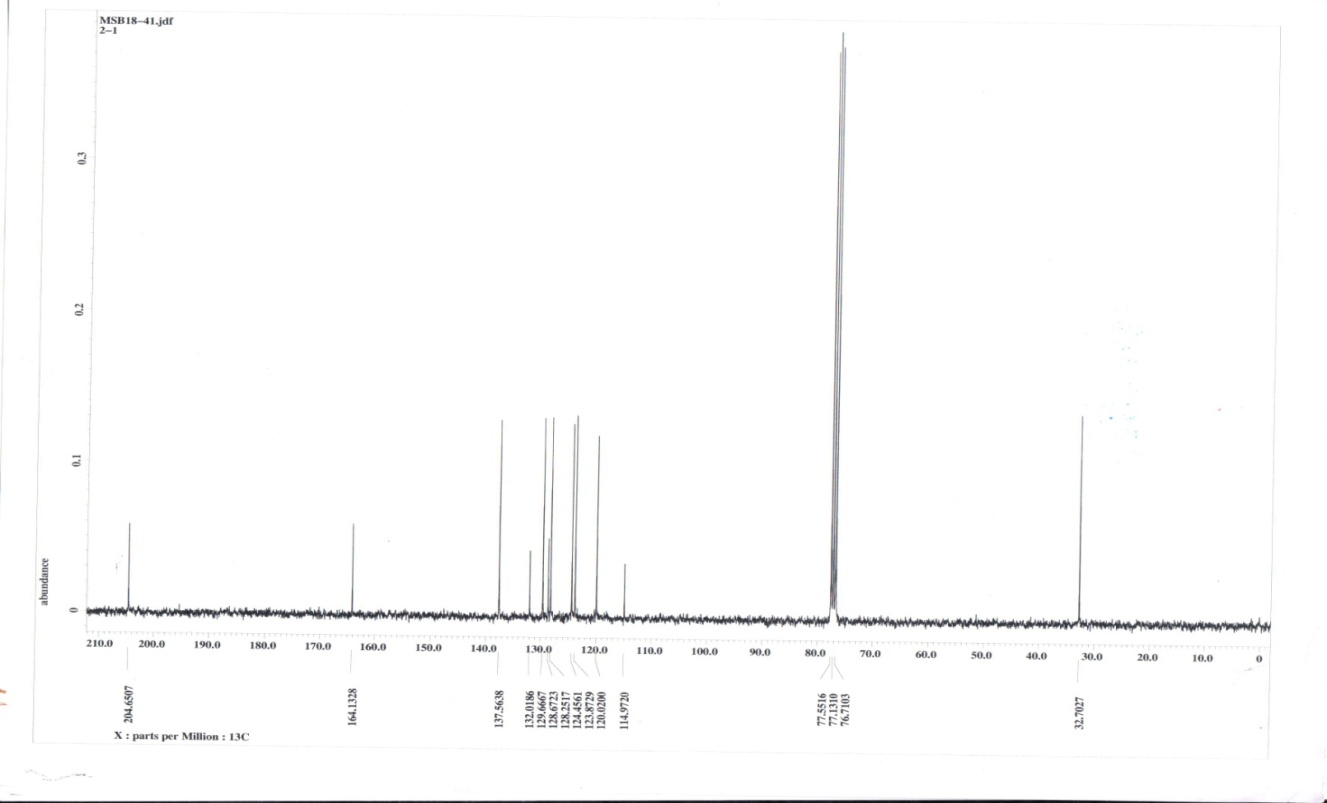


**Supplementary figure 2** ^13^C NMR spectra of (*S*)-1-(2-Hydroxy-1-naphthyl) ethanol


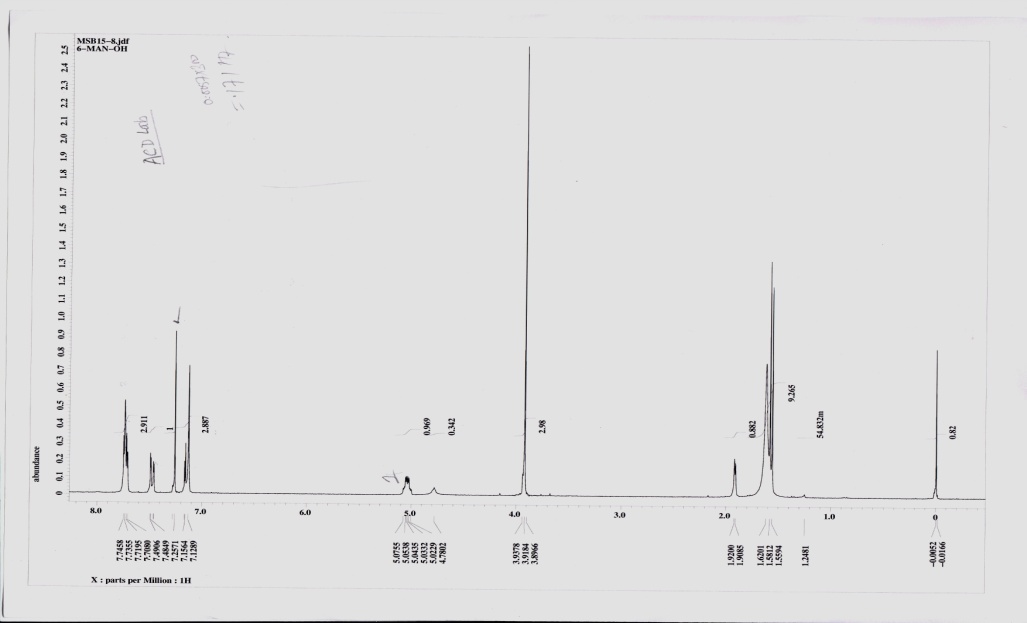


**Supplementary figure 3** ^1^H NMR spectra of 1-(6-methoxy-2-naphthyl) ethanol


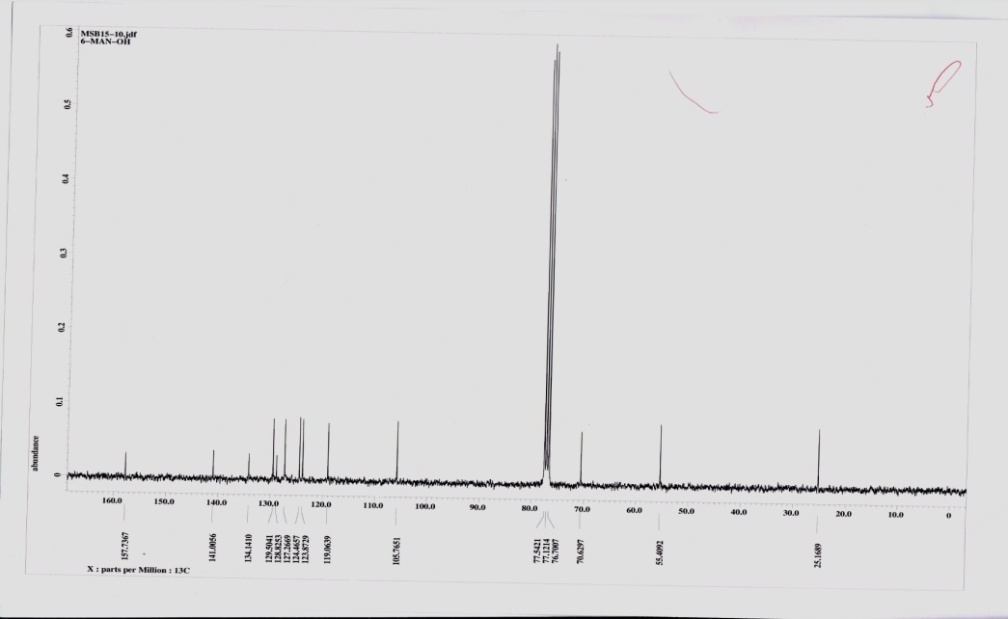


**Supplementary figure 4** ^13^C NMR spectra of 1-(6-methoxy-2-naphthyl) ethanol


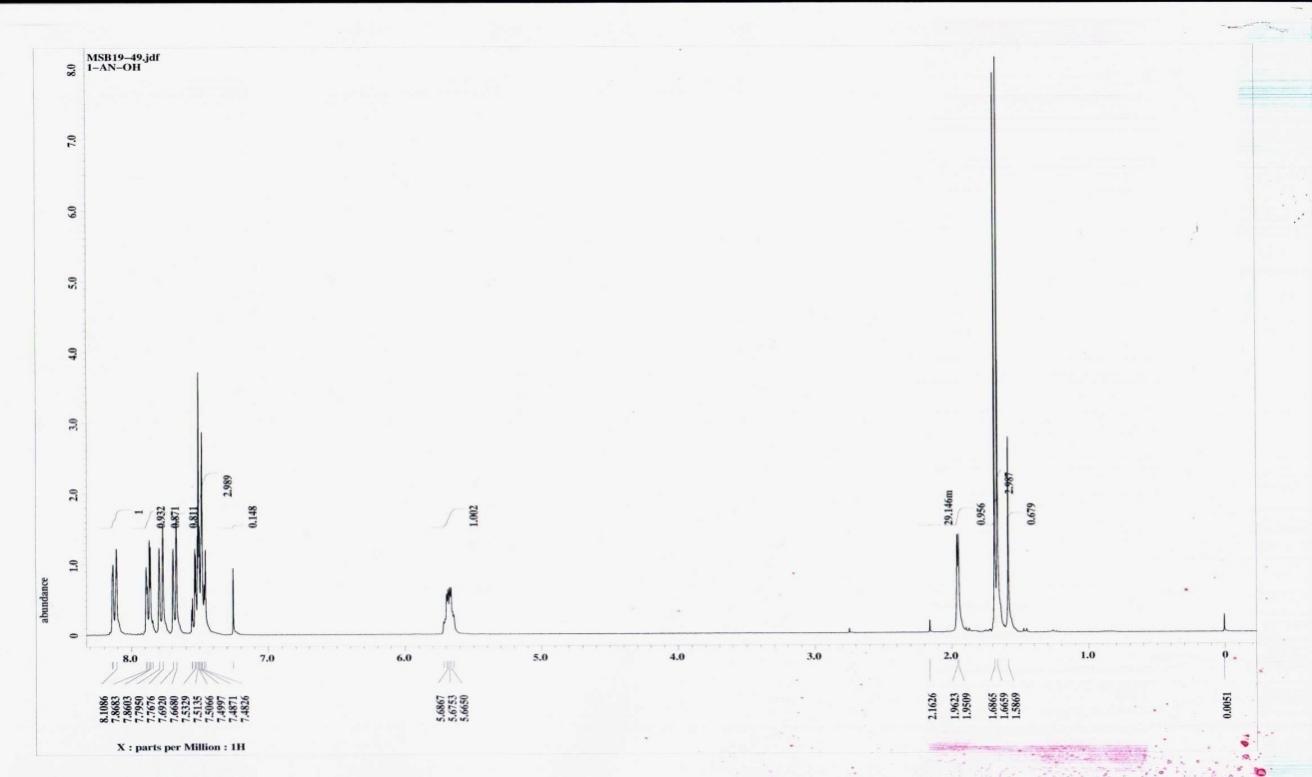


**Supplementary figure 5** ^1^H NMR spectra of (S)-(−)-1-(1-Naphthyl) ethanol


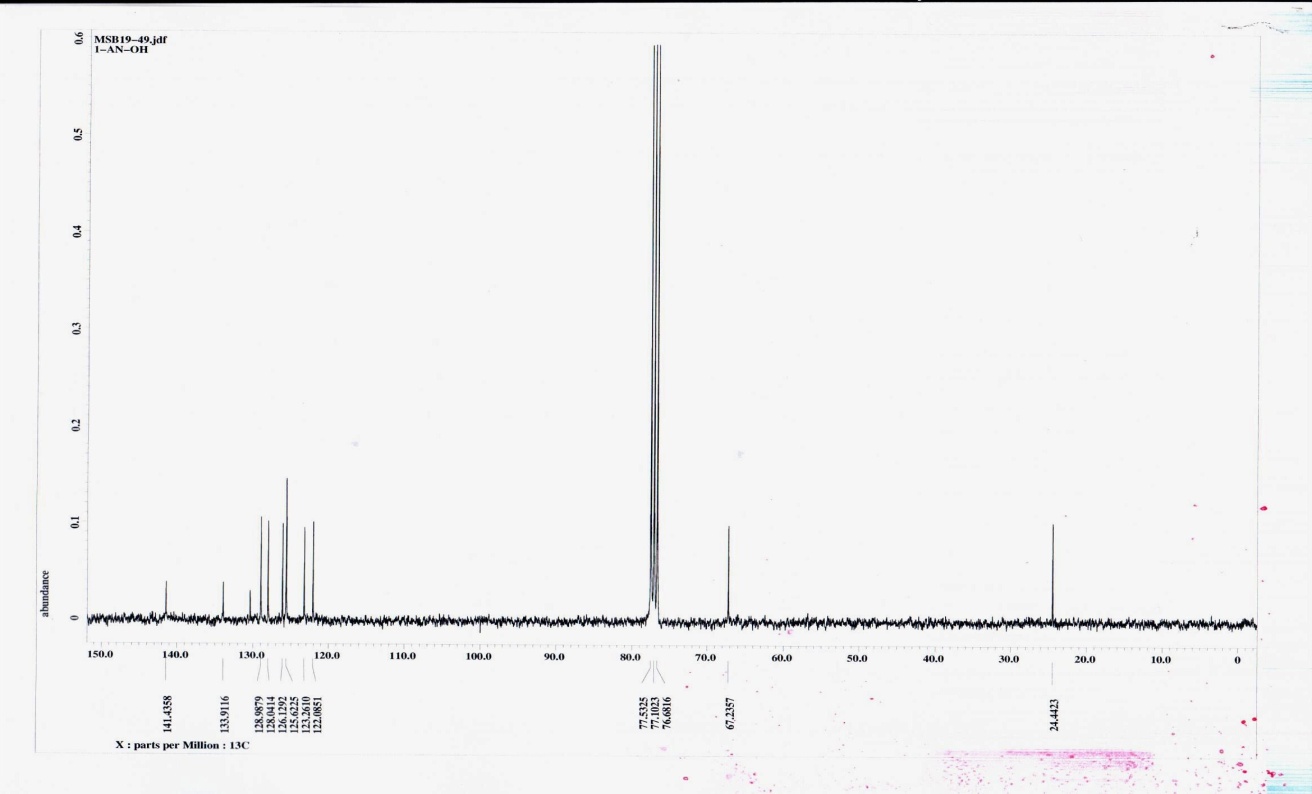


**Supplementary figure 6** ^13^C NMR spectra of (S)-(−)-1-(1-Naphthyl) ethanol

A

B

**Supplementary figure 7.** Qualitative analysis of biosurfactant produced by HSB-15^T^ strain: (**A**) oil displacement assay and (**B**) thin layer chromatography of produced biosurfactant the spot C1 corresponds to a reference standard sophorolipid. The spots C2 and C3 represent biosurfactant samples from two different batches.


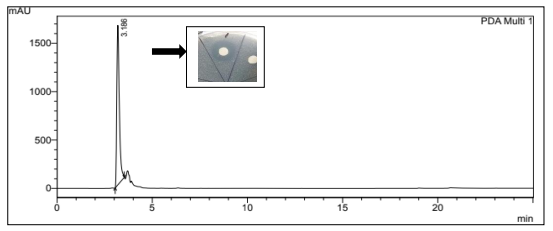


**Supplementary figure 8 .** HPLC chromatogram showing peak of biosurfactant produced from HSB-15^T^ strain having RT of 3.16 minutes. The antimicrobial fraction was identified by testing it against *S. aureus* cells in a agar plate by observing the zone of inhibition. The purified fraction was applied for antibacterial activity against *S. aureus* cells and a clear zone of inhibition can be seen (indicated by arrow).


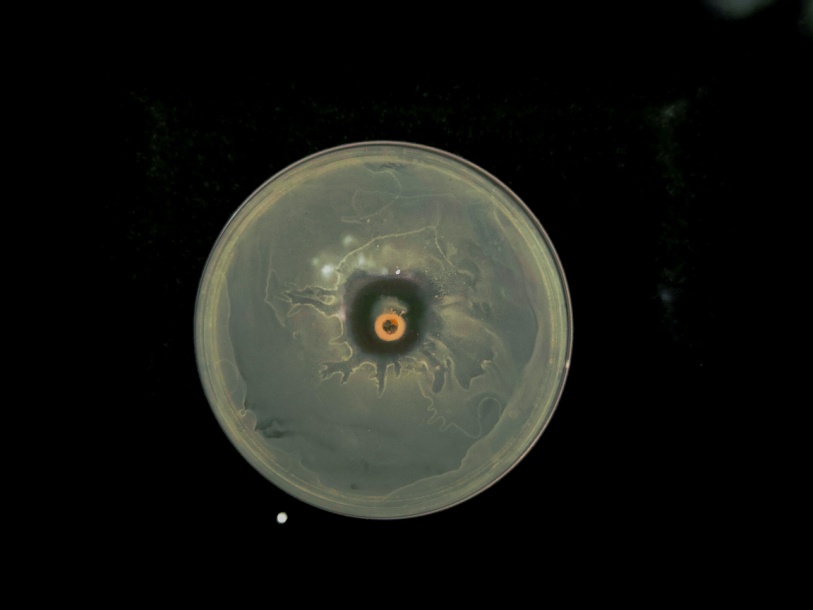


**Supplementary figure 9.** Image of zone of inhibition in *S. aureus* cells. About 100 µL of the HPLC purified sample (500µg/mL) was added in LB agar plate containing *S. aureus* cells. After incubation of the plate at 37 °C overnight, a clear zone of inhibition was observed and found to be 20mm in diameter.
